# Mother’s Pre-pregnancy BMI and Placental Candidate miRNAs: Findings from the ENVIR*ON*AGE Birth Cohort

**DOI:** 10.1038/s41598-017-04026-8

**Published:** 2017-07-17

**Authors:** Maria Tsamou, Dries S. Martens, Ellen Winckelmans, Narjes Madhloum, Bianca Cox, Wilfried Gyselaers, Tim S. Nawrot, Karen Vrijens

**Affiliations:** 1 Center for Environmental Sciences, Hasselt University, Diepenbeek, Belgium; 2 Department of Obstetrics, East-Limburg Hospital, Genk, Belgium; 3 Department of Public Health, Environment & Health Unit, Leuven University (KU Leuven), Leuven, Belgium

## Abstract

There is increasing evidence that the predisposition for development of chronic diseases arises at the earliest times of life. In this context, maternal pre-pregnancy weight might modify fetal metabolism and the child’s predisposition to develop disease later in life. The aim of this study is to investigate the association between maternal pre-pregnancy body mass index (BMI) and miRNA alterations in placental tissue at birth. In 211 mother-newborn pairs from the ENVIR*ON*AGE birth cohort, we assessed placental expression of seven miRNAs important in crucial cellular processes implicated in adipogenesis and/or obesity. Multiple linear regression models were used to address the associations between pre-pregnancy BMI and placental candidate miRNA expression. Maternal pre-pregnancy BMI averaged (±SD) 23.9 (±4.1) kg/m^2^. In newborn girls (not in boys) placental miR-20a, miR-34a and miR-222 expression was lower with higher maternal pre-pregnancy BMI. In addition, the association between maternal pre-pregnancy BMI and placental expression of these miRNAs in girls was modified by gestational weight gain. The lower expression of these miRNAs in placenta in association with pre-pregnancy BMI, was only evident in mothers with low weight gain (<14 kg). The placental expression of miR-20a, miR-34a, miR-146a, miR-210 and miR-222 may provide a sex-specific basis for epigenetic effects of pre-pregnancy BMI.

## Introduction

Detrimental effects of a disturbed intrauterine environment, caused by maternal inadequate nutrition before and during pregnancy, on fetal growth and the development of metabolic disease in adult life were first reported by Barker^1^. Prevalence of obesity is increasing worldwide, posing a high risk for pregnancy complications^2^. *In utero* exposure to maternal obesity has a key role in adverse pregnancy outcomes (pre-eclampsia, gestational diabetes, gestational hypertension, infant mortality and prematurity), fetal development (large for gestational age, congenital anomalies) and metabolic programming^3, 4^. Particularly, a maternal obesogenic environment has been associated with increased systolic blood pressure in offspring^5^, development of schizophrenia, elevated levels of cholesterol and triglycerides, impaired glucose tolerance, obesity and cardiovascular risk in childhood^2, 6^ and in adulthood^7^. Induction of oxidative stress and chronic inflammation in newborns^8, 9^ and children^10^ has been linked to maternal obesity during pregnancy. There is increasing evidence that *in utero* exposures can influence birth outcomes via epigenetic mechanisms including microRNA (miRNA) expression^11^. MiRNAs can regulate expression of up to one-third of the human genome^12^ and are involved in many physiological processes influencing adverse health outcomes in later life, including obesity and diabetes^11^. As such, miRNAs are implicated in adipocyte differentiation, metabolic integration, insulin resistance and appetite regulation, all of which are relevant for the onset and development of obesity^13^.

Numerous studies have focused on the role of miRNAs in pregnancy complications, including pre-eclampsia, prematurity and gestational diabetes mellitus^14^. Although several miRNAs have been linked to BMI in adults^15, 16^, evidence on the association between maternal pre-pregnancy BMI and fetal miRNA expression is very limited.

We selected a set of seven miRNAs previously shown to be expressed in human placental tissue or cell lines (Table 1), all of which are known to be related to crucial processes involved in the ontogeny or maintenance of obesity, such as inflammation^17^, oxidative stress^18^, apoptosis^19^, cell cycle^20^ and angiogenesis^21^: miR-16^22^, miR-20a^23^, miR-21^24^, miR-34a^25^, miR-146a^26^, miR-210^27^ and miR-222^28^ (Table 5).

**Table 1:** Relevant studies of the studied candidate miRNAs on human placental tissue or cell lines.

miRNA	Function	Pathologic or Physiologic condition/Exposure	Biological system	Regulation	Reference
miR-16	Cell cycle & apoptosis	Development	Placenta	+BW, −SGA & −NAS	66, 85
		Pre-eclampsia	Placenta	−	67
		Cigarrete smoke	Placenta	+	37
miR-20a	Cell proliferation & invasion, angiogenesis	Pre-eclampsia	Placenta & BeWo cells	+	68
		Pre-eclampsia	JEG-3 cells	+	69
		Trimester-specific PM_2.5_	Placenta	+/−	65
miR-21	Cell cycle & proliferation	Development	Placenta	+BW & −SGA	70, 85
		Cigarrete smoke	Placenta	−	37
		Trimester-specific PM_2.5_	Placenta	+/−	65
miR-34a	Cell growth & invasion	Placenta accreta	JAR cells & placenta	−	63
		Cervical cancer	BeWo & JAR cells	+	71
		Pre-eclampsia	Placenta	−	86
miR-146a	Inflammation	Development	Placenta	+MS	66
		Pre-eclampsia	Placenta	−	68
		BSA	Trophoblast cells	+	72
		Nicotine&BaP/Cigarrete smoke	TCL-1 cells & placenta	−	37
		Trimester-specific PM_2.5_	Placenta	−	65
miR-210	Hypoxia/Oxidative stress	Preeclampsia	Placenta	+	73
miR-222	Angiogenesis	Pre-eclampsia	Placenta	+	67
		Gestational Diabetes	Placenta	−	74
		Trimester-specific PM_2.5_	Placenta	−	65

BW: birth weight, NAS: neonatal attention scores, SGA: small gestational age, BeWo, JEG-3 & JAR cells: trophoblast choriocarcinoma cell lines, TCL-1 cells: extravillous trophoblast from choriodecidua of a term placenta, MS: movement scores, BSA: bisphenol A, BaP: Benzopyrene.

We hypothesize that maternal pre-pregnancy BMI affects newborn’s epigenetic changes through differential miRNA expression in placental tissue at birth, which may provide further insights into mechanisms underlying these potential associations.

## Results

### General characteristics of the study population

Demographic characteristics of 211 mother-newborn pairs are given in Table 2. The mothers had an average (±SD) age of 29.5 (±4.3) years and an average (±SD) maternal pre-pregnancy BMI of 23.9 (±4.1) kg/m^2^. 49 (23.2%) of the mothers were overweight and 19 (9.0%) were obese, while 6 (2.8%) were underweight. Based on the Institute of Medicine (IOM) recommendations^29^ on the maternal gestational weight gain, 44.5% (n = 94) had an excessive weight gain, while 38.4% (n = 81) had a normal weight gain. The newborns, among them 112 girls (53.1%), had a mean (range) gestational age of 39.2 (35–41) weeks and comprised of 103 (48.8%) primiparous and 85 (40.3%) secundiparous newborns. The mean (±SD) birth weight of the newborns was 3413 (±447) grams. About 90.1% (n = 190) of the newborns were Europeans of Caucasian ethnicity and 3.3% delivered by Caesarian section (C-section). The average (±SD) gestational weight gain was 14.9 (±5.8) kg. Most of the mothers (71.1%) never smoked cigarettes and 57.8% had a high educational level.

**Table 2:** Demographic characteristics of study population (n = 211).

Characteristics	Mean ± SD/Frequency (%)		
	Both genders (n = 211)	Boys (n = 99)	Girls (n = 112)
**Maternal**			
*Age*, years	29.5 ± 4.3	29.2 ± 4.3	29.7 ± 4.2
*Pre*-*gestational weight*, kg	66.5 ± 13.3	66.9 ± 11.8	66.2 ± 14.5
*Height*, m	1.67 ± 0.1	1.67 ± 0.1	1.67 ± 0.1
*Pre*-*pregnancy BMI*, kg/m^2^	23.9 ± 4.1	24.1 ± 3.7	23.7 ± 4.4
*Gestational weight gain*, kg	14.9 ± 5.8	14.7 ± 5.3	15.1 ± 6.2
*BMI at delivery*, kg/m^2^	29.3 ± 4.5	29.5 ± 3.8	29.1 ± 5.1
*Smoking status*			
Never-smoker	150 (71.1)	68 (68.7)	82 (73.2)
Past-smoker	31 (14.7)	14 (14.1)	17 (15.2)
Current- smoker	30 (14.2)	17 (17.2)	13 (11.6)
*Parity*			
1	103 (48.8)	49 (49.5)	54 (48.2)
2	85 (40.3)	38 (38.4)	47 (42.0)
≥3	23 (10.9)	12 (12.1)	11 (9.8)
*Education*			
Low	24 (11.4)	11 (11.1)	13 (11.6)
Middle	65 (30.8)	38 (38.4)	27 (24.1)
High	122 (57.8)	50 (50.5)	72 (64.3)
*Gestational diabetes*	5 (2.7)	3 (3.0)	2 (1.8)
*Hypertension*	5 (2.7)	2 (2.0)	3 (2.7)
*Hyper/Hypothyroidism*	4 (1.9)	1 (1.0)	3 (2.7)
*Pre*-*eclampsia*	2 (0.9)	—	2 (1.8)
*Asthma*	2 (0.9)	1 (1.0)	1 (0.9)
*Gastric band*	3 (1.4)	2 (2.0)	1 (0.9)
**Newborn**			
*Gestational age*, weeks	39.2 ± 1.3	39.1 ± 1.4	39.3 ± 1.2
*Birth weight*, g	3,413 ± 447	3,466 ± 476	3,366 ± 416
*Ethnicity*			
European-Caucasian	190 (90.1)	87 (87.9)	103 (92.0)
Non-European	21 (9.9)	12 (12.1)	9 (8.0)
*C*-*section*	7 (3.3)	5 (5.1)	2 (1.8)
**Other**			
*Outdoor Temperature*, °C			
Third trimester (quartiles)			
<5.6	51 (24.2)	23 (23.2)	28 (25.0)
≥5.6 and <9.4	54 (25.6)	23 (23.2)	31 (27.7)
≥9.4 and <14.8	52 (24.6)	27 (27.3)	25 (22.3)
≥14.8	54 (25.6)	26 (26.3)	28 (25.0)

Within our study population, two pregnant women (0.9%) had a diagnosis of pre-eclampsia, five mothers (2.7%) were diagnosed with gestational diabetes, five mothers (2.7%) had hypertension, four (1.9%) had hyper- or hypothyroidism and two mothers (0.9%) had asthma. Three mothers (1.4%) underwent gastric banding surgery for weight loss prior to their pregnancy.

### Association of maternal pre-pregnancy BMI, gestational weight gain and BMI at delivery with placental miRNA expression at birth

Placental relative expression of miRNAs did not differ between girls and boys, did not correlate with maternal age, gestational duration, parity and maternal education. To allow for potential non-linear associations between placental miRNA expression and outdoor temperature, we categorized temperature based on quartiles and found that miRNA expression was positively correlated with low outdoor temperature.

Independent of newborn’s ethnicity and gestational age, maternal age, smoking status, educational status, parity, gestational weight gain, health complications, delivery by C-section and outdoor temperature during third trimester, placental expression miR-20a (−5.85%, [CI: −10.91, −0.50, *p* = *0*.*035*]), miR-34a (−8.85%, [CI: −15.22, −2.00, *p* = *0*.*014*]) and miR-222 (−4.64%, [CI: −9.23, 0.18, *p* = *0*.*062*]) were lower with increasing maternal pre-pregnancy BMI in newborn girls but not in newborn boys (Table 3).

**Table 3:** Changes (%) in placental relative miRNA expression associated with maternal pre-pregnancy BMI, gestational weight gain and BMI at delivery.

miRNAs	Pre-pregnancy BMI^a^		Gestational weight gain^b^		BMI at delivery^b^	
	% change (95% CI)	*P*-value	% change (95% CI)	*P*-value	% change (95% CI)	*P*-value
*Girls* (*n* = *112*)						
miR-16	−4.26 (−9.28, 1.05)	0.12	0.57 (−3.11, 4.39)	0.77	−2.98 (−7.27, 1.51)	0.19
miR-20a	−5.85 (−10.91, −0.50)	0.035	1.21 (−2.57, 5.13)	0.54	−3.87 (−8.25, 0.72)	0.10
miR-21	−4.65 (−10.69, 1.80)	0.16	0.98 (−3.48, 5.65)	0.67	−3.08 (−8.26, 2.39)	0.27
miR-34a	−8.85 (−15.22, −2.00)	0.014	−1.45 (−6.14, 3.46)	0.56	−7.90 (−13.32, −2.15)	0.009
miR-146a	−4.77 (−9.76, 0.50)	0.078^†^	−2.60 (−6.92, 1.93)	0.26^¥^	−3.40 (−7.72, 1.13)	0.14
miR-210	−4.95 (−11.19, 1.72)	0.14^†^	−4.15 (−9.48, 1.49)	0.15^¥^	−4.64 (−9.94, 0.97)	0.11
miR-222	−4.84 (−9.38, −0.09)	0.049^†^	−1.99 (−5.95, 2.12)	0.34^¥^	−3.43 (−7.34, 0.65)	0.10
*Boys* (*n* = *99*)						
miR-16	0.20 (−6.35, 7.21)	0.95	2.73 (−2.21, 7.91)	0.29	0.99 (−5.36, 7.76)	0.77
miR-20a	−2.99 (−9.57, 4.07)	0.40	2.31 (−2.80, 7.69)	0.38	−2.10 (−8.49, 4.74)	0.54
miR-21	−2.58 (−9.85, 5.28)	0.51	0.06 (−5.44, 5.89)	0.98	−2.61 (−9.55, 4.87)	0.48
miR-34a	4.01 (−5.34, 14.29)	0.42	2.03 (−4.26, 8.73)	0.54	3.81 (−5.09, 13.55)	0.42
miR-146a	−1.97 (−8.14, 4.62)	0.55	0.80 (−3.87, 5.70)	0.74	−1.74 (−7.66, 4.56)	0.58
miR-210	−3.81 (−11.65, 4.71)	0.37	5.45 (−0.89, 12.19)	0.097	−1.61 (−9.44, 6.91)	0.70
miR-222	−0.76 (−6.76, 5.63)	0.81	3.85 (−0.77, 8.69)	0.11	0.84 (−5.08, 7.13)	0.79

Estimates (95% confidence intervals) for a 1 kg/m^2^ increase in BMI or for a 1 kg increment in gestational weight gain.

^a^Adjusted for newborn’s ethnicity and gestational age, maternal age, smoking status, educational status, parity, gestational weight gain, health complications, delivery by C-section and outdoor temperature during the third trimester of pregnancy.

^b^Adjusted for same set of covariates except gestational weight gain.

^†^Additionally adjusted for the interaction between pre-pregnancy BMI and gestational weight gain. Estimate for a gestational weight gain of 14 kg (50^th^ percentile).

^¥^Additionally adjusted for the interaction between pre-pregnancy BMI and gestational weight gain. Estimate for a pre-pregnancy BMI of 23 kg/m^2^ (50^th^ percentile).

**Figure 1:**
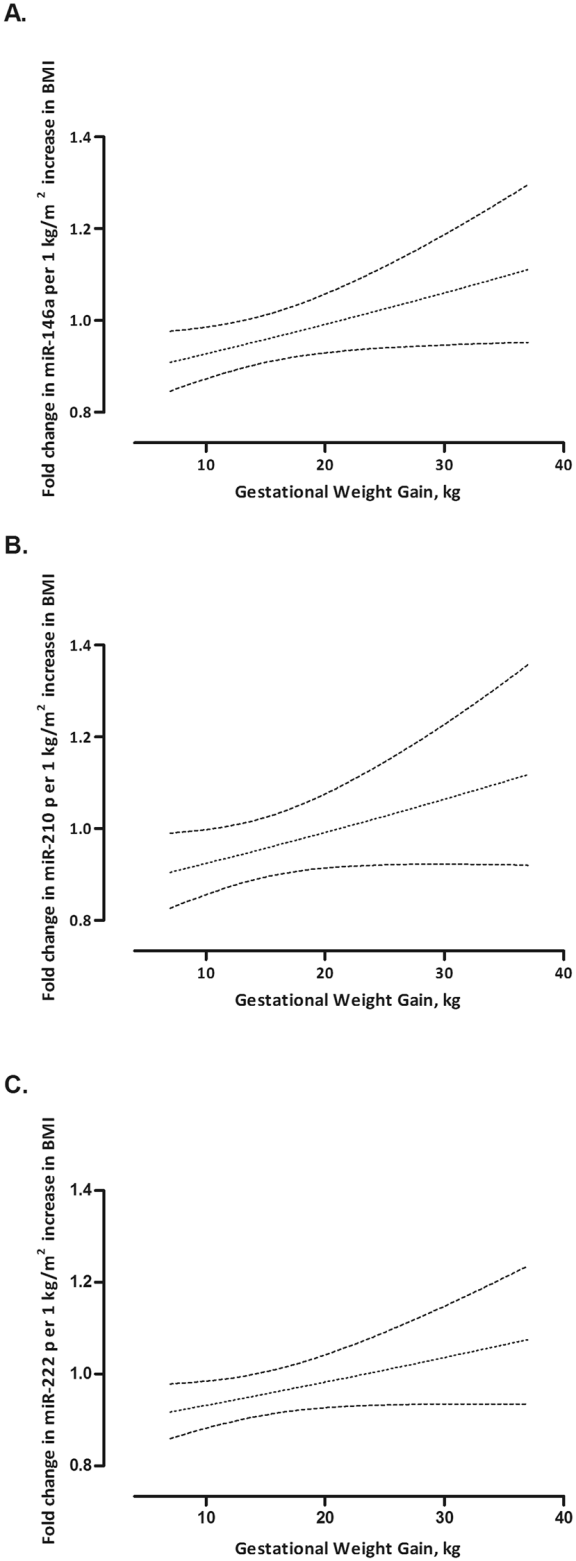
Effect modification by gestational weight gain on the association between maternal pre-pregnancy BMI and placental expression of miR-146a (panel A), miR-210 (panel B) and miR-222 (panel C) in newborn girls.

With similar adjustments as before, we explored the interaction between pre-pregnancy BMI and weight gain during pregnancy using continuous nature of the variables. These interaction terms were not significant for miR-16, miR-20a, miR-21 and miR-34a, but borderline significant (*p*_*interaction*_ < *0*.*10*) for miR-210 and miR-222, and significant for miRNA-146a (*p* = *0*.*043*), only in girls. In addition, in girls the associations between pre-pregnancy BMI and placental miRNAs (miR-146a, miR-210 and miR-222) which were found to be modified by the gestational weight gain are illustrated in Figure 1. Placental miR-146a (−6.66%, [CI: −11.92, −1.09, *p* = *0*.*022*]), miR-210 (−6.94%, [CI: −13.50, 0.12, *p* = *0*.*057*) and miR-222 (−6.34%, [CI: −11.14, −1.29, *p* = *0*.*016*]) expression was inversely associated with each increase of 1 unit (1 kg/m^2^) in mother’s pre-pregnancy BMI for a gestational weight gain of 11 kg (corresponding to 25^th^ percentile). The association was not significant in mothers with a gestational weight gain above approximately 14 kg. Table 4 provides the predicted effect sizes, for an increase in pre-pregnancy BMI for 1 kg/m² and for 7, 11, 14, 18 and 25 kg increase in gestational weight gain. These adjusted estimates accounted for the aforementioned covariates and include the interaction between pre-pregnancy BMI and gestational weight gain.

**Table 4:** Changes (%) in placental miRNA (miR-146a, miR-210 and miR-222) expression in newborn girls in association with pre-pregnancy BMI at specific gestational weight gain values.

Gestational Weight Gain (kg)^a^	miR-146a		miR-210		miR-222	
	% change (95% CI)	*P*-value	% change (95% CI)	*P*-value	% change (95% CI)	*P*-value
7	−9.12 (−15.40, −2.38)	0.01	−9.53 (−17.34, −0.98)	0.032	−8.31 (−14.07, −2.16)	0.01
11	−6.66 (−11.92, −1.09)	0.022	−6.94 (−13.50, 0.12)	0.057	−6.34 (−11.14, −1.29)	0.016
14	−4.77 (−9.76, 0.50)	0.078	−4.95 (−11.19, 1.72)	0.14	−4.84 (−9.38, −0.09)	0.049
18	−2.19 (−7.74, 3.70)	0.46	−2.23 (−9.18, 5.24)	0.55	−2.81 (−7.82, 2.48)	0.29
25	2.50 (−5.99, 11.76)	0.58	2.70 (−7.90, 14.53)	0.63	0.86 (−6.74, 9.08)	0.83

Estimates (95% confidence intervals) are given for a 1 kg/m^2^ increase in BMI and adjusted for newborn’s ethnicity and gestational age, maternal age, smoking status, educational status, parity, gestational weight gain, interaction between pre-pregnancy BMI and gestational weight gain, health complications, delivery by C-section and outdoor temperature during third trimester.

^a^The gestational weight gain (kg) is indicated for 5^th^, 25^th^, 50^th^, 75^th^ and 95^th^ percentiles.

Considering maternal BMI at delivery, only the placental relative miR-34a expression was associated with mother’s BMI at delivery (−7.90% for 1 unit BMI increase, [CI: −13.32, −2.15, *p* = *0*.*009*]).

Finally, sensitivity analyses, from which we excluded mothers with pregnancy complications, or with C-section deliveries were confirmatory (see Supplementary Table S1).

## Discussion

Maternal obesity signifies an important public health issue, as it affects the health of the mother and has a large impact on the health of the unborn fetus possibly leading to health risks later in life^10, 30–32^. The onset of metabolic diseases including obesity in childhood or adulthood has been described to commence in early life^33^. Perturbations in the intrauterine environment can affect epigenetic mechanisms, which are involved in fetal programming and could be important in development of various diseases throughout life^34^.

MiRNAs play key regulatory roles in diverse biological processes, including inflammation, oxidative stress, apoptosis, angiogenesis, adipogenesis, which are all implicated in development of metabolic disorders, such as obesity and cardiovascular diseases^13, 35, 36^.

Our results indicate that higher maternal pre-pregnancy BMI is associated with lower placental miRNA expression for miR-20a, miR-34a, miR-146a, miR-210 and miR-222 expression in girls but not in boys. Further, the effect of pre-pregnancy BMI on some placental miRNA (miR-146a, miR-210 and miR-222) expression was modified by maternal gestational weight gain.

Our study population consisted mostly (64.9%) of women with normal pre-pregnancy BMI (23.9 ± 4.1 kg/m^2^) and found that for 1 kg/m^2^ increment in pre-pregnancy BMI corresponds to a decrease in placental miRNA expression within a range of 4.3–8.8%, in girls. These changes for only 1 unit increase in pre-pregnancy BMI were similar to the range of decrease (3.9–9.4%) in miRNA expression, including miR-16, miR-21 and miR-146a, reported for placentas exposed to maternal cigarette smoke during pregnancy^37^.

Aberrant expression of miR-222 has been reported in obese or morbidly obese men compared to a control group of healthy weight individuals (n = 80). Decreased levels of circulating miR-16 and increased levels of miR-21 and miR-146a were found in morbidly obese patients (n = 22) before versus after surgically-induced weight loss^22^. Higher placental expression of miR-210 has been shown in obese mothers (pre-pregnancy BMI > 30 kg/m^2^) versus mothers with a healthy weight (n = 36)^38^. In addition, overexpression of miR-20a^23^, miR-21^24^, miR-34a^39^, and decreased expression of miR-210 and miR-222^39^ has been linked to increased adipocyte differentiation.

The observed changes in placental candidate miRNA expression associated with an increase in maternal pre-pregnancy BMI may regulate processes involved in placental inflammation and angiogenesis. Increased levels of placental pro-inflammatory cytokines have been reported in response to maternal obesity^40, 41^. Maternal obesity has been also linked to placental abruption and abnormal spiral artery remodeling which is likely to be caused by insufficient trophoblast invasion^42^. MiR-146a has been involved in inflammatory responses by activating NF-kB signaling^43^. Decreased levels of miR-146a expression in serum were linked to chronic inflammation in diabetic patients^26^, insulin resistance and pro-inflammatory cytokine genes^44^. Moreover, Dentelli *et al*., have reported that down-modulated miR-222 in endothelial cells was involved in inflammation-mediated vascular remodeling^45^.

Based on numerous studies, many miRNAs also referred to as angiomiRs have been identified to play an important role in (placental) angiogenesis^46, 47^. Among them, miR-21, miR-20a and miR-210 have been shown to induce angiogenesis, whereas miR-16, miR-34a and miR-222 inhibit angiogenesis (Table 5). In placenta, miR-20a and miR-34a have been identified in spiral artery remodeling, miR-16 in vascular endothelial growth factor signaling pathway and miR-210 in trophoblastic migration and invasion^47^. In case of an imbalance between pro- and anti-angiogenic factors, an increased or decreased formation of blood vessels can occur^48^, which may lead to abnormal placental development^47^. Additionally, oxidative stress has been implicated in placental vascular dysfunction^49^.

**Table 5:** Measured miRNAs related to key processes implicated in obesity.

miRNA	Regulation	Function	Biological system	Target	Reference
miR-16	↑	Inhibition of blood vessel formation & migration of trophoblast cells	Human Umbilical Vein Endothelial Cells	↓*CCNE1* & *VEGFA*	75
	↓	Weight loss in severe obesity (surgery-induced)	Human Plasma		22
miR-20a	↑	Inhibition of spheroid cell sprouting, network formation & cell migration	Human Endothelial Cells		76
	↑	Adipogenesis	Mouse Adipocytes (3T3L1)	↓*Rb2*	23
miR-21	↑	Inhibition of cell proliferation, migration & tubulogenesis	Human Umbilical Vein Endothelial Cells	↓*RhoB*	77
	↑	Adipogenesis	Human Adipose tissue-derived mesenchymal Stem Cells (hASCs)	↓*TGFBR2*	24
	↑	Weight loss in severe obesity (surgery-induced)	Human Plasma		22
miR-34a	↑	Inhibition of angiogenesis by induction of senescence	Rat Endothelial Progenitor Cells	↓*SIRT1*	78
	↓	Reduced adiposity	Murine White/Brown Adipose Tissue	↑*FGF21* & *SIRT1*	25
	↑	Adipogenesis	Human mature adipocytes		39
miR-146a	↑	Weight loss in severe obesity (surgery-induced)	Human Plasma		22
	↑	Increased cell migration, tube formation & angiogenesis	Human Umbilical Vein Endothelial Cells	↓*CARD10* & *NF-kB*	79
	↓	Inflammation/ Increased insulin resistance	Human peripheral blood mononuclear cells (Diabetic patients)/ Human Serum	↑*TNFα/*↑*IL*-*8* & *HGF*	26
miR-210	↓	Decreased tubulogenesis & cell migration (normoxic conditions)	Human Umbilical Vein Endothelial Cells		80
	↑	Enhanced angiogenesis	Human endothelial cells		36
	↓	Adipogenesis	Human mature adipocytes		39
	↑	Maternal Obesity	Human placenta (girls)	↑*TNFα*	38
miR-222	↑	Reduced tube formation, migration & wound healing	Human Umbilical Vein Endothelial Cells	↓*KIT* & *eNOS*	81, 82
	↑	Inhibition cell migration	Human Umbilical Vein Endothelial Cells	↓*ETS1*	83
	↓	Inflammation-mediated vascular growth factors	Human Endothelial Cells	↑*STAT5a*	45
	↓	Adipogenesis	Human mature adipocytes		39
	↑	Severe obesity	Human Plasma (men or children)		22, 84

*CARD10*: Caspase Recruitment Domain Family, Member 10, *CCNE1*: Cyclin E1, *eNOS*: endothelial Nitric Oxide Synthase 3, *ETS1*: V-Ets Avian Erythroblastosis Virus E26 Oncogene Homolog 1, *FGF21*: Fibroblast growth factor 21, *HGF*: Hepatocyte growth factor, *IL*-*8*: Interleukin 8, *NF*-*kB*: Nuclear Factor Of Kappa Light Polypeptide Gene Enhancer In B-Cells, *KIT*: V-Kit Hardy-Zuckerman 4 Feline Sarcoma Viral Oncogene Homolog, *Rb2*: Retinoblastoma-like protein 2, *RhoB*: Ras Homolog Family Member B, *SIRT1*: Sirtuin 1, *STAT5a*: Signal transducer and activator of transcription 5A, *TGFBR2*: Transforming Growth Factor, Beta Receptor II, *TNFa*: Tumor necrosis factor a, *VEGFA*: Vascular endothelial growth factor A.

We analyzed the association between placental miRNA expression and maternal pre-pregnancy BMI in a gender-specific approach and found no association with our miRNA candidates and mothers pre-pregnancy BMI in boys. This is in line with a previous smaller study (n = 36)^38^ that noted only significant alterations in placental miRNA expression in association with mother’s pre-pregnancy BMI for girls. This supports the hypothesis that placental miRNAs in female fetuses are more responsive to maternal BMI changes than those from boys. Several studies have shown that fetal development and growth exhibit gender specificity^50–52^. Gender differences have been observed when studying placental immune function^53^, gene^54^ and protein expression^53^.

Till now, the exact mechanism underlying this observed sex-specificity on placenta remains unknown. The increased sensitivity of the female placenta observed here, as well as in other studies^51, 55–57^, might be interpreted as potential protective mechanisms, that might explain the lower risk for adult diseases, such as hypertension and/or cardiovascular diseases, in women compared with men^58, 59^.

A strong point of our study is the use of measured pre-pregnancy BMI at the first antenatal visit of the mother at the hospital (around week 7–9 of pregnancy) that minimizes the chance of misreported pre-pregnancy BMI. In addition, there is no large population based birth cohort yet which addressed the association between pre-pregnancy BMI and placental miRNA expression. Notwithstanding these strong points, our study should be interpreted within the context of its possible limitations. First, the obesity-related factors can induce epigenetic changes in germ line cells as well, that can be transmittable to the next generation. In this regard, a study showed that paternal obesity modulates sperm miRNA content and germ cell methylation and impairs the metabolic status of the next generation^60^. We did not have information of the BMI of the father. Second, based only on these findings we cannot assure whether these miRNA alterations persist in later life. Finally, we adjusted our statistical model for a range of possible covariates, but this does not rule out the possibility of under- or over-estimation by other important variables not yet identified which may be associated with both maternal pre-pregnancy BMI and placental miRNA expression.

## Conclusion

We observed an inverse association between maternal pre-pregnancy BMI and placental miRNA expression in a sex-specific pattern, with effects of placental miR-20a, miR-34a, miR-146a, miR-210 and miR-222 only in newborn girls. These miRNAs may be implicated in the development of metabolic diseases in postnatal or later life. Therefore, we believe maintaining a healthy BMI before pregnancy is an important factor to contribute to normal placental function and can prevent detrimental health outcomes later in life. Further studies are needed to investigate long-term effects of these molecular changes.

## Methods

### Study population

This study included 215 mother-newborn pairs (with only singletons) selected from the ongoing ENVIR*ON*AGE (ENVIRonmental influence ON AGEing in early life) birth cohort in the province Limburg in Belgium^61^. The study protocol was approved by the Ethical Committee of Hasselt University and South-East-Limburg Hospital (ZOL) in Genk (Belgium) and has been carried out according to the declaration of Helsinki. Written informed consent was obtained from all participating mothers. The recruitment of mother-newborn pairs took place from March 2010 till January 2014, between Friday 12.00 hours and Monday 07.00 hours. Inclusion criteria included the mother’s ability to fill out questionnaires in Dutch. The overall participation rate was 61%. Detailed information on maternal age, education, occupation, smoking status, alcohol consumption, place of residence, use of medication, parity and newborn’s ethnicity were provided by the participants. Smoking status of the mothers was categorized into three groups: non-smokers (those who never smoked), past-smokers (those who quit smoking before pregnancy) and current-smokers (those who continued smoking during pregnancy). Ethnicity of the newborn was classified based on the native country of the newborn’s grandparents: European-Caucasian (those with more than two European grandparents) and non-European (those with at least three non-European grandparents). Maternal educational status was grouped as low (no diploma or primary school), middle (high school) or high (college or university degree).

The maternal health complications including pre-eclampsia, gestational diabetes, hypertension, hypo- or hyperthyroidism, asthma, gastric band surgery prior to pregnancy, were retrieved from the medical records.

Data on mean daily outdoor temperature for the study region were provided by the Royal Meteorological Institute (Brussels, Belgium).

### Maternal pre-pregnancy BMI, gestational weight gain and BMI at delivery

The maternal pre-pregnancy BMI was recorded in the hospital at the first antenatal visit around week 7–9 of pregnancy. Maternal height and weight were measured to the nearest centimeter and to the nearest 0.1 kg respectively, without wearing shoes and wearing light clothes. Maternal pre-pregnancy BMI is calculated as the pre-pregnancy body weight (kg) divided by the square of the height (m^2^). According to the World Health Organization (WHO) classification^62^, BMI in adults is categorized into the following groups: underweight (<18.5 kg/m^2^), normal weight (18.5–24.9 kg/m^2^), overweight (25.0–29.9 kg/m^2^) and obese (≥30 kg/m^2^). Women with a pre-pregnancy BMI > 40 kg/m^2^ (class III obesity) were excluded from further analyses, resulting in a final study population of 211 mother-newborn pairs.

The gestational weight gain (kg) is defined as the weight at delivery minus the pre-pregnancy weight. Mothers were weighed on admission to the delivery ward to obtain their weight at delivery. Using the recommended gestational weight gain guidelines by the IOM^29^, based on the maternal pre-pregnancy BMI according to WHO^62^, the gestational weight gain is expected to be within the range of 12.7–18.1 kg for underweight, 11.3–15.9 kg for normal weight, 6.8–11.3 kg for overweight and 5.0–9.1 kg for obese women. The BMI (kg/m^2^) at delivery is calculated by the weight (kg) at delivery divided by the square of the height (m^2^).

### Selection candidate miRNAs

In this study, we investigated the effect of maternal pre-pregnancy BMI on placental candidate miRNA expression, using the fetal portion of placental tissue. We selected a set of seven miRNAs, miR-16, miR-20a, miR-21, miR-34a, miR-146a, miR-210 and miR-222, which are expressed in human placental tissue and are involved in key cellular processes (Table 1 and Table 5) implicated in obesity^15, 17, 18^. MiR-16 has been implicated in regulation of the cell cycle and apoptosis, miR-20a in cell proliferation, invasion and angiogenesis, miR-21 in cell cycle and proliferation, miR-34a in cell growth and invasion, miR-146a in inflammation, miR-210 in angiogenesis and oxidative stress, and miR-222 in angiogenesis. Furthermore, the selected miRNAs for study are known to be responsive to various *in utero* exposures which possibly affect fetal programming and subsequently can lead to adverse health outcomes in later life^37, 63–65^.

### Sample Collection

After delivery, placental tissue was collected and deep-frozen within 10 minutes. On the fetal side of the placenta, four standardized biopsies were collected and stored in RNA later at 4 °C overnight and then at −20 °C for longer period. These biopsies were taken at fixed locations across the middle point of the placenta, at approximately 4 cm distance from the umbilical cord.

### RNA isolation and DNase treatment

Total RNA and miRNA were extracted from pooled placenta biopsies using the miRNeasy mini kit (Qiagen, KJ Venlo, the Netherlands) according to the manufacturer’s protocol. We used pooled placenta samples from 4 collected biopsies, in order to minimize possible intra-placental variation. Quantity and purity of the extracted total RNA and miRNA was assessed by spectrophotometry (Nanodrop ND-1000; Isogen Life Science, De Meern, the Netherlands). The average (±SD) yield of total RNA per placenta pooled biopsies was 4.4 (±1.2) µg with average A_260/280_ and A_260/230_ ratios of 1.95 (±0.04) and 1.71 (±0.16), respectively. DNase treatment was performed on extracted RNA samples according to the manufacturer’s instructions (Turbo DNA-free kit, Ambion, Life Technologies, Diegem, Belgium). Isolated RNA was stored at −80 °C until further applications.

### Reverse transcription and miRNA expression analysis

Briefly, using the TaqMan miRNA Reverse Transcription Kit (Applied Biosystems, Foster City, CA) and Megaplex stem-loop primer pool A (Applied Biosystems, Foster City, CA), RNA was reverse transcribed allowing miRNA specific cDNA synthesis, which we performed for the 7 selected miRNAs, according to the manufacturer’s protocol. All target sequences of the miRNAs and control RNA are available in Supplementary Table S2. RNA was reverse transcribed and produced cDNA was stored at −20 °C for a maximum of one week until further downstream measurements. For the measurement of miRNA expression, cDNA was used for PCR reactions on a 7900HT Fast Real-Time PCR System (Applied Biosystems, Foster City, CA), using Taqman miRNA assays (Applied Biosystems, Foster City, CA), all according to the manufacturer’s protocol. Detailed methods have been described previously^65^. For normalization the endogenous control RNU6 was used. In order to minimize the technical variation between the different runs of the same miRNA assay, inter-run calibrators (IRCs) were applied. Amplification efficiencies were between 90–115% for all assays. The obtained Cq values were extracted by SDS 2.3 software (Applied Biosystems, Foster City, CA) and then the relative miRNA expression was calculated by 2^−ΔΔCq^ method using qBase plus software (Biogazelle, Belgium). All samples were analyzed in triplicate. Replicates were included when ΔC_q_ was smaller than 0.5.

### Statistical analysis

The relative quantities of miRNA expressions were log-transformed (log10) because of their non-normal distribution. Maternal pre-pregnancy BMI, gestational weight gain and BMI at delivery were included as continuous variables. SAS software (Version 9.4 SAS Institute, Cary, NC, USA) was used for the statistical analysis.

The associations between log10-transformed relative placental miRNA expression and maternal pre-pregnancy BMI, gestational weight gain and BMI at delivery were assessed using multiple linear regression models, stratifying by gender (n = 112 girls, n = 99 boys). Models were adjusted for the following *a priori* chosen covariates: newborn’s ethnicity (European or non-European) and gestational age (weeks), maternal age (years), smoking status (never-smoker, past-smoker or current-smoker), educational status (low, middle or high), parity (1, 2 or ≥3), health complications (pre-eclampsia, gestational diabetes, hypertension, hypo/hyperthyroidism, asthma and gastric band surgery prior to pregnancy), delivery by C-section and outdoor temperature during the third trimester of pregnancy (categorized into quartiles). Maternal health complications were treated as dichotomous variables. Models with pre-pregnancy BMI as independent variable were additionally adjusted for gestational weight gain.

We started by testing the interaction between pre-pregnancy BMI and gestational weight gain for each measured miRNA. The interaction term was kept in the final model when the p-value was <0.1, and the estimated effect of pre-pregnancy BMI (or gestational weight gain) was reported for the median value of gestational weight gain (or pre-pregnancy BMI).

We calculated the change (%) in placental miRNA expression per 1 unit increase in the independent variable of interest (1 kg/m^2^ for pre-pregnancy BMI and BMI at delivery; 1 kg for gestational weight gain) as follows:

$$Change\,( \% )=(({1}{{0}}^{\beta }-{1})\ast {100}),$$

with 95% confidence intervals (CI), where *β* is the estimated regression coefficient.

## Electronic supplementary material

Supplementary Information
