# Author Correction: Mother’s Pre-pregnancy BMI and Placental Candidate miRNAs: Findings from the ENVIR*ON*AGE Birth Cohort

**DOI:** 10.1038/s41598-018-24090-y

**Published:** 2018-04-11

**Authors:** Maria Tsamou, Dries S. Martens, Ellen Winckelmans, Narjes Madhloum, Bianca Cox, Wilfried Gyselaers, Tim S. Nawrot, Karen Vrijens

**Affiliations:** 1 Center for Environmental Sciences, Hasselt University, Diepenbeek, Belgium; 2 Department of Obstetrics, East-Limburg Hospital, Genk, Belgium; 3 Department of Public Health, Environment & Health Unit, Leuven University (KU Leuven), Leuven, Belgium

Correction to: *Scientific Reports* 10.1038/s41598-017-04026-8, published online 17 July 2017

This Article contains errors in Table 1.

For the miR-16, the Pre-eclampsia regulation should read ‘+’, and the Cigarette smoke regulation should read ‘−’ respectively.

In addition for miR-222, the row entitled ‘Gestational Diabetes’ should be removed respectively.

In addition, this Article contains errors in the Introduction.

“We selected a set of seven miRNAs previously shown to be expressed in human placental tissue or cell lines (Table 1), all of which are known to be related to crucial processes involved in the ontogeny or maintenance of obesity, such as inflammation, oxidative stress, apoptosis, cell cycle and angiogenesis: miR-16, miR-20a, miR-21, miR-34a, miR-146a, miR-210 and miR-222 (Table 5).”

should read:

“We selected a set of seven miRNAs previously shown to be expressed in human placental tissue or cell lines (Table 1), all of which are known to be related to crucial processes involved in the ontogeny or maintenance of obesity, such as inflammation, oxidative stress, apoptosis, cell cycle and angiogenesis: miR-16, miR-20a, miR-21, miR-34a, miR-146a, miR-210 and miR-222.”

