# Supplementary material for: Mother’s Pre-pregnancy BMI and Placental Candidate miRNAs: Findings from the ENVIRONAGE Birth Cohort
Source: Sci Rep. 2017 Jul 17;7:5548. doi: 10.1038/s41598-017-04026-8 (PMC5514037; doi:10.1038/s41598-017-04026-8)
Supplement: Supplementary file 1 — Supplementary Information [file 41598_2017_4026_MOESM1_ESM.doc]

**SUPPLEMENTARY MATERIAL to:**

***Mother’s Pre-pregnancy BMI and Placental Candidate miRNAs: Findings from the ENVIRONAGE Birth Cohort***

**Authors**: Maria Tsamou^1^, Dries S Martens^1^, Ellen Winckelmans^1^, Narjes Madhloum^1^, Bianca Cox^1^, Wilfried Gyselaers^2^, Tim S Nawrot^1,3^†, Karen Vrijens^1^†

†these authors contributed equally to this work

**Affiliations:**

^1^Center for Environmental Sciences, Hasselt University, Diepenbeek, Belgium;

^2^Department of Obstetrics, East-Limburg Hospital, Genk, Belgium;

^3^Department of Public Health, Environment & Health Unit, Leuven University (KU Leuven), Leuven, Belgium;

***Supplementary Table S1. Changes (%) in placental relative miRNA expression associated with maternal pre-pregnancy BMI, per each increase of 1 unit in BMI (kg/m^2^).***

|  | **Main Analysis^a^** |  | **Sensitivity Analysis^a^** | | | | | |
| --- | --- | --- | --- | --- | --- | --- | --- | --- |
| **miRNAs** | **Pre-pregnancy BMI** |  | **Excluded maternal health complications** |  | **Excluded C-sections** |  | **Excluded maternal health complications and C-sections** | |
|  | **% change (95% CI)** | ***P-*value** | **% change (95% CI)** | ***P-*value** | **% change (95% CI)** | ***P-*value** | **% change (95%CI)** | ***P-***  **value** |
| **Girls** | ***n=112*** |  | ***n=102*** |  | ***n=110*** |  | ***n=100*** |  |
| **miR-16** | -4.26 (-9.28, 1.05) | 0.12 | -4.08 (-9.29, 1.42) | 0.15 | -4.28 (-9.32, 1.04) | 0.12 | -4.10 (-9.33, 1.43) | 0.15 |
| **miR-20a** | -5.85 (-10.91, -0.50) | 0.035 | -5.39 (-10.73, 0.26) | 0.064 | -5.93 (-10.87,-0.72) | 0.028 | -5.48 (-10.67, 0.01) | 0.054 |
| **miR-21** | -4.65 (-10.69, 1.80) | 0.16 | -3.68 (-9.57, 2.60) | 0.25 | -4.69 (-10.73, 1.75) | 0.15 | -3.72 (-9.62, 2.56) | 0.24 |
| **miR-34a** | -8.85 (-15.22, -2.00) | 0.014 | -9.33 (-15.71, -2.46) | 0.01 | -8.92 (-15.22, -2.16) | 0.012 | -9.40 (-15.67, -2.65) | 0.009 |
| **miR-146a†** | -4.77 (-9.76, 0.50) | 0.078 | -4.57 (-9.91, 1.09) | 0.11 | -4.85 (-9.76, 0.33) | 0.069 | -4.65 (-9.91, 0.91) | 0.1 |
| **miR-210†** | -4.95 (-11.19, 1.72) | 0.14 | -4.12 (-10.45, 2.66) | 0.23 | -5.08 (-11.16, 1.42) | 0.13 | -4.25 (-10.40, 2.32) | 0.21 |
| **miR-222†** | -4.84 (-9.38, -0.09) | 0.04 | -4.18 (-8.85, 0.73) | 0.098 | -4.92 (-9.37, -0.25) | 0.042 | -4.26 (-8.84, 0.53) | 0.084 |
|  |  |  |  |  |  |  |  |  |
| **Boys** | ***n=99*** |  | ***n=90*** |  | ***n=94*** |  | ***n=85*** |  |
| **miR-16** | 0.20 (-6.35, 7.21) | 0.95 | -0.94 (-8.11, 6.79) | 0.81 | 0.30 (-6.45, 7.54) | 0.93 | -0.94 (-8.11, 6.79) | 0.81 |
| **miR-20a** | -2.99 (-9.57, 4.07) | 0.4 | -4.92 (-12.21, 2.98) | 0.22 | -3.72 (-10.32, 3.36) | 0.3 | -4.92 (-12.21, 2.98) | 0.22 |
| **miR-21** | -2.58 (-9.85, 5.28) | 0.51 | -4.21 (-12.19, 4.50) | 0.33 | -2.86 (-10.30, 5.20) | 0.48 | -4.21 (-12.19, 4.50) | 0.33 |
| **miR-34a** | 4.01 (-5.34, 14.29) | 0.42 | -3.40 (-7.22, 15.23) | 0.55 | 4.12 (-5.49, 14.70) | 0.42 | -3.40 (-7.22, 15.23) | 0.55 |
| **miR-146a** | -1.97 (-8.14,4.62) | 0.55 | -3.47 (-10.21, 3.78) | 0.34 | -2.24 (-8.49, 4.43) | 0.5 | -3.47 (-10.21, 3.78) | 0.34 |
| **miR-210** | -3.81 (-11.65, 4.71) | 0.37 | -7.41 (-15.74, 1.74) | 0.11 | -4.49 (-12.39, 4.14) | 0.3 | -7.41 (-15.74, 1.74) | 0.11 |
| **miR-222** | -0.76 (-6.76, 5.63) | 0.81 | -1.26 (-8.12, 6.12) | 0.73 | -0.85 (-6.97, 5.67) | 0.79 | -1.26 (-8.12, 6.12) | 0.73 |

*Estimates (95% confidence intervals) in miRNA expression with all participants (in main analysis), without those who had health complications before or during pregnancy and/or delivered by C-section (sensitivity analysis).*

*^a^ Adjusted for newborn’s ethnicity and gestational age, maternal age, smoking status, educational status, parity, gestational weight gain, health complications, delivery by C-section and outdoor temperature during third trimester.*

†*Adjusted for interaction between pre-pregnancy BMI and gestational weight gain , for a gestational weight gain of 14kg (50^th^ percentile).*

**Supplementary Table S2. TaqMan microRNA assay numbers and target sequences for all miRNAs and control used.**

| **Assay ID** | **Gene Symbol** | **Assay Target Sequence** | **miRBase ID or NCBI Name** |
| --- | --- | --- | --- |
| **000391** | hsa-miR-16 | -UAGCAGCACGUAAAUAUUGGCG- | hsa-miR-16-5p |
| **000580** | hsa-miR-20a | -UAAAGUGCUUAUAGUGCAGGUAG- | hsa-miR-20a-5p |
| **000397** | hsa-miR-21 | -UAGCUUAUCAGACUGAUGUUGA- | hsa-miR-21-5p |
| **000426** | hsa-miR-34a | -UGGCAGUGUCUUAGCUGGUUGU- | hsa-miR-34a-5p |
| **000468** | hsa-miR-146a | -UGAGAACUGAAUUCCAUGGGUU- | hsa-miR-146a-5p |
| **000512** | hsa-miR-210 | -CUGUGCGUGUGACAGCGGCUGA- | hsa-miR-210-3p |
| **002276** | hsa-miR-222 | -AGCUACAUCUGGCUACUGGGU- | hsa-miR-222-3p |
| **001973** | U6 snRNA | -GUGCUCGCUUCGGCAGCACAUAU  ACUAAAAUUGGAACGAUACAGAGA  AGAUUAGCAUGGCCCCUGCGCAAG  GAUGACACGCAAAUUCGUGAAGCG  UUCCAUAUUUU- | U6 snRNA |
